# Recombinant Human Perlecan DV and Its LG3 Subdomain Are Neuroprotective and Acutely Functionally Restorative in Severe Experimental Ischemic Stroke

**DOI:** 10.1007/s12975-022-01089-2

**Published:** 2022-12-12

**Authors:** Ifechukwude Joachim Biose, Ibolya Rutkai, Bryan Clossen, Gary Gage, Kenneth Schechtman, H. Davis Adkisson, Gregory J. Bix

**Affiliations:** 1 Department of Neurosurgery, Clinical Neuroscience Research Center, Tulane University School of Medicine, New Orleans, LA 70112 USA; 2 Tulane Brain Institute, Tulane University, New Orleans, LA 70112 USA; 3 Stream Biomedical, Inc., 2450 Holcombe, Suite J, Houston, TX 77021 USA; 4 Division of Biostatistics, Washington University School of Medicine, St. Louis, MO 63110 USA; 5 Department of Neurology, Tulane University School of Medicine, New Orleans, LA 70112 USA

**Keywords:** Perlecan, Perlecan domain V, LG3, Basement membrane, Neuroprotection and acute ischemic stroke

## Abstract

Despite recent therapeutic advancements, ischemic stroke remains a major cause of death and disability. It has been previously demonstrated that  ~ 85-kDa recombinant human perlecan domain V (rhPDV) binds to upregulated integrin receptors (α2β1 and α5β1) associated with neuroprotective and functional improvements in various animal models of acute ischemic stroke. Recombinant human perlecan laminin-like globular domain 3 (rhPDV_LG3_), a 21-kDa C-terminal subdomain of rhPDV, has been demonstrated to more avidly bind to the α2β1 integrin receptor than its parent molecule and consequently was postulated to evoke significant neuroprotective and functional effects. To test this hypothesis, fifty male C57Bl/6 J mice studied in a t-MCAO model were randomly allocated to either rhPDV treatment, rhPDV_LG3_, or equivalent volume of PBS at the time of reperfusion in a study where all procedures and analyses were conducted blind to treatment. On post-MCAO day 7, *2,3,5-triphenyltetrazolium chloride* staining of brain slices was used to quantify infarct volume. We observed that treatment with rhPDV_LG3_ reduced infarct volume by 65.6% (*p* = 0.0001), improved weight loss (*p* < 0.05), and improved functional outcome measures (*p* < 0.05) when compared to PBS controls, improvements which were generally greater in magnitude than those observed for 2 mg/kg of rhPDV. In addition, treatment with 6 mg/kg of rhPDV_LG3_ was observed to significantly reduce mortality due to stroke in one model, an outcome not previously observed for rhPDV. Our initial findings suggest that treatment with rhPDV_LG3_ provides significant improvement in neuroprotective and functional outcomes in experimental stroke models and that further investigation of rhPDV_LG3_ as a novel neuroprotective therapy for patients with stroke is warranted.

## Introduction

Stroke is one of the leading causes of death and permanent disability across the globe [1, 2]. Approximately every 40 s, a patient in the USA experiences a stroke, resulting in an annual economic burden for the USA alone of over $45 billion [2]. Approximately 87% of all stroke cases are due to ischemic stroke [2]. Large vessel occlusion (LVO) is the most disabling form of stroke; approximately half of ischemic stroke cases in the USA involves LVO [3]. Importantly, LVO stroke carries the highest mortality burden when compared to non-LVO stroke for aged adults (31.1% vs 4.6%, respectively). Timely restoration of cerebral blood flow (CBF) is the only established effective treatment for ischemic stroke through either mechanical thrombectomy intervention and/or thrombolysis. Unfortunately, significant disability often remains even after reperfusion by thrombolysis or thrombectomy, and these therapeutic modalities are often associated with adverse events, including reperfusion injury and hemorrhagic transformation that may further increase brain damage and disability [4–6]. Therefore, despite recent advances in the clinical management of acute ischemic stroke, there remains a considerable need for safe and efficacious neuroprotective treatments.

Previous work has demonstrated the efficacy of recombinant perlecan domain V (rhPDV), an ~ 85-kDa C-terminal protein fragment of vascular basement membrane proteoglycan, in reducing infarct volume and functional deficits following transient proximal and distal middle cerebral artery occlusion (t-MCAO, d-MCAO) in young and aged mice [7, 8]. In addition, it has been demonstrated that rhPDV displays differential binding kinetics to both α2β1 and α5β1 integrins, demonstrating twofold greater avidity to α2β1 integrin [9, 10]. Given the mechanistic role with which inside-out integrin signaling has historically promoted cell survival, proliferation, and/or the production of trophic factors, selectivity and binding of the c-terminal fragment (laminin-like globular domain 3 (LG3)) of perlecan domain V (DV) within the functional neurovascular unit may be associated with increased neuroprotective properties.

The basal lamina of the cerebral vasculature is enriched in perlecan, a highly conserved modular, multifunctional heparan sulfate proteoglycan recognized to preserve the integrity of tissue borders including that of the heart and brain. LG3 is one of the three laminin G-like modules comprising the c-terminal region of domain V. During acute cerebral ischemia, perlecan content of the infarct core is abruptly (2 h) disrupted via proteolysis [11], and this change in extracellular matrix (ECM) composition is associated with neuronal injury [12]. Bix and colleagues have shown that the soluble peptide, perlecan DV, is rapidly and persistently elevated within the ECM of stroked rodent and human brains [6, 13]. Given the time at which this molecule is normally generated in vivo and the robust neuroprotection associated with its parenteral administration, rhPDV is believed to represent a novel stroke therapy offering both neuroprotective and neuroregenerative benefits when administered in supraphysiologic concentrations. Unpublished in vitro results from Bix and colleagues suggest that recombinant human perlecan LG3 (rhPDV_LG3_), a 21-kDa subdomain of rhPDV, may confer the biological activity of parent DV with potentially greater affinity to select integrins. Hitherto, the neuroprotective effect of immediate rhPDV treatment has only been tested in the context of moderate stroke models (e.g. d-MCAO). Here, we sought to expand the investigation of the effects of rhPDV, and more specifically rhPDV_LG3_, in the context of t-MCAO, a more severe form of ischemic stroke which is experimentally modeled by occluding the origin of the MCA by intraluminal monofilament or suture [14].

Prior to selecting DV constructs to be utilized in the severe stroke model studies, we performed a 3-day screening study to examine the in vivo bioactivity of rhPDV_LG3_ and multiple other rhPDV constructs utilizing the highly consistent distal (d-MCAO) stroke model. This model provides an ideal opportunity for testing neuroprotective efficacy in vivo, as it provides for visual confirmation of occlusion (including location and success) and reperfusion, highly consistent infarcts (in both vascular territory and overall volume), few to no functional deficits, and no mortality [13, 15]. Taken together, these characteristics eliminate the majority of confounding variables inherent to other stroke models and present an ideal model for studies focused on neuronal loss and protective mechanisms without the need for significant functional deficits.

Multiple rhPDV constructs (including LG3) were screened using d-MCAO; the DV constructs were administered at the max dose (and associated max infarct volume reduction) reported by the Bix Lab [13] (2 mg/kg), and the dose producing the max infarct volume reduction for LG3 in this screening model (6 mg/kg) was determined. Based on the initial findings of the d-MCAO model, we selected those doses that provided equivalent neuroprotection for investigation in a 7-day severe stroke recovery study to assess therapeutic properties associated with rhPDV_LG3_ (utilizing previously studied DV_HEK293_ recombinant human full-length perlecan DV as the reference standard) [13].

## Materials and Methods

### Animals

For the 3-day neuroprotection screening study, a total of forty-eight male C57Bl/6 J mice (10–12 weeks old, 24–30 g, The Jackson Laboratory, USA) were randomly allocated to eight groups: rhPDV_HEK293_ (1 or 2 mg/kg), rhPDV_Gly_ (2 mg/kg), rhPDV_NQ_ (2 mg/kg), rhPDV_LG3_ (2, 4, or 6 mg/kg), or equivalent volume of PBS vehicle control (*n* = 5–7 per group). For the 7-day severe ischemic stroke recovery study, a total of fifty male C57Bl/6 J mice (10–12 weeks old, 24–30 g, The Jackson Laboratory, USA) were randomly allocated to three groups: (i) PBS vehicle control (*n* = 15), (ii) rhPDV group (*n* = 16), and (iii) rhPDV_LG3_ group (*n* = 19). All mice were maintained in identical housing conditions: no more than five mice per cage and standard 12 h light–dark cycle along with unlimited access to water and feed. A minimum of one week was allowed for acclimatization before experimentation from the date of mouse delivery to Stream’s housing facility at STILLMEADOW, Inc. or Tulane’s animal housing facilities.

### Preparation for Middle Cerebral Artery Occlusion

All mice were subjected to aseptic surgical technique for the induction of 60 min transient occlusion of either the proximal or distal middle cerebral artery (MCA) per the intraluminal filament model Koizumi et al. (modified for mice) [16] or the tandem common carotid artery and middle cerebral artery transient occlusion model of Aronowski et al., respectively [15, 17].

Anesthetic induction was achieved with isoflurane carried in oxygen and maintained at 1–1.5% during surgical procedures via a nose cone. The absence of hindlimb pinch reflex confirmed adequate anesthesia. Eye ointment (Artificial tears, Akorn Inc., USA) was applied to both eyes to prevent corneal desiccation. The fur over the surgical sites of the ventral neck and above or behind the left zygomatic arch was removed by depilatory cream (Nair™, Church and Dwight Co Inc., USA) before transfer to the surgical table with a heating pad to maintain normal body temperature (36.5–37.5 °C, temperature controller, Harvard Apparatus, USA). Prior to incision, the skin was disinfected using alcohol and betadine, and ropivacaine block (1–2 mg/kg s.c., NDC17478-081–30, Akorn Operating Company LLC, USA) was administered at the sites of incision.

### Tandem Common Carotid-Middle Cerebral Artery Transient Occlusion (Distal/d-MCAO)

Using Nair™, hair was removed from the center neck region from the chest to chin (~ 0.75″) and the left temporal head region from the eye to ear (~ 0.5″). One milligram of buprenorphine SR per kilogram body weight was then administered subcutaneously for pre-emptive analgesia. A midline incision was made in the ventral neck and left common carotid artery (CCA) elevated with a 6–0 suture. The incision was briefly closed, and the subject rotated such that an incision could be made along the ridge of the temporalis muscle. A small burr hole was then made through the skull, above the visible distal branch of the middle cerebral artery (MCA). A fine steel wire was run through the dura, under the MCA, and back out of the skull such that the elevation point occluded the MCA, and cessation of blood flow was visually confirmed. The CCA was then temporarily ligated with a suture, and the subject was placed in a recovery cage for the 60-min occlusion period. At that time, the CCA ligation was removed, and then the elevating wire under the MCA was removed, with reperfusion being visually confirmed prior to closure of the incisions. Following closing of both incisions, subjects were returned to a heated recovery cage until bright, alert, and reactive (BAR) status achieved.

All subjects were inspected for complete wound closure and placed in a heated recovery cage under an infrared heating lamp for 30 min to 1 h. After checking that the animals had regained mobility, subjects were returned to its home cage. Post-operation monitoring occurred twice daily, with body weight measured prior to surgery, and again every day up to 72 h. Lack of BAR status, refusal to eat, sustained significant weight loss, and abnormal lethargy were considered unacceptable and exclusionary criteria that would qualify a subject for early termination. No adverse events or post-operative issues were observed in any animal.

### Intraluminal Filament Transient MCAO (t-MCAO)

Pre-occlusion blood flow in the MCA territory was measured, using laser Doppler flowmetry (PeriFlux system 5000, Perimed AB, Sweden) by placing the single-point laser probe just above the zygomatic arch between the left eye and ear. An incision in the ventral neck and careful separation of the salivary glands was made to expose the common carotid artery (CCA). The external (ECA) and internal (ICA) carotid arteries were isolated superior to the CCA. Using 5–0 silk suture, ligatures were placed at the proximal portion of the CCA (permanent knot) and another (loose knot) below CCA bifurcation but above the ventral cut to prevent blood loss. A 6–0 nylon silicone-coated tip monofilament (602356PK5Re, Doccol Corporation, MA, USA) was inserted into the ventral cut on the CCA and advanced through the ICA and past the base of the skull into the circle of Willis to occlude the origin of the MCA. Each mouse was recovered from anesthesia in a pre-warmed cage for 60 min prior to reperfusion. Upon reperfusion of the MCA, the monofilament was removed to re-establish blood flow after 60 min of occlusion. Cerebral perfusion was assessed prior to and following MCAO induction. Only animals with > 60% reduction of pre-occlusion values were included in the study.

A splash block of the neck muscles (with 1–2 mg/kg of ropivacaine) was administered prior to the closure of the neck and head incisions using 7-mm wound clips (CellPoint Scientific Inc., USA). Normal saline (0.5 mL, ICU Medical Inc., USA) and 1–2 mg/kg of ropivacaine were subcutaneously administered immediately after reperfusion and daily for 3 days post-surgery for hydration and analgesia, respectively. Additionally, moistened food pellets and soft diet (DietGel® 76A, ClearH_2_O, USA) were provided in home cages, for the study duration, to encourage feeding and hydration. Mice were observed twice daily and weighed once daily following t-MCAO surgery to monitor welfare, health, and activity. We determined, a priori, to euthanize any mouse found unable to rise on all four limbs due to significant welfare concern.

### Expression of Perlecan Domain V Constructs

Full length rhPDV was expressed in Chinese hamster ovary cells (CHO K1) using a proprietary cytomegalovirus vector under the control of human elongation factor-1 alpha (pCI-EF1) promoter. Plasmids contained a signal sequence upstream of the start of the DV sequence that was separated by a “glycine kinker.” Human cDNA encoding the native sequence, containing 3 putative N-glycosylation sites, was inserted in the first construct designated rhPDV_Gly_. Given the unknown role with which glycosylation modulates the neuroprotective function in perlecan DV, a second construct of the same length was created in which the Asn residue in the three consensus N-linked glycosylation sites was mutated to Gln (N-Q mutations; rhPDV_NQ_). Each construct contained a COOH-terminal 8 × HIS tag to facilitate protein purification. CHO K1 cells were grown as recommended by the manufacturer (Thermo Fisher Scientific, Waltham, MA) and transfected using the Lipofectamine reagent. Post-transfection cells were grown at 32 °C, 5% CO_2_, and pH 7.15 and batch fed at 12 h and again at day 5, with the final harvest occurring between days 10 and 14 of culture. HIS-tagged protein was enriched using a two-column chromatography process. rhPDV_HEK293_ utilized as the reference standard was expressed in human kidney embryonic cells as described previously and similarly contained a c-terminal HIS tag to facilitate enrichment [13].

Due to the lack of glycosylation sites in human LG3, rhPDV_LG3_ was expressed in *E. coli* [BL21(DE3)] utilizing an expression plasmid driven by the T7 polymerase promoter, without addition of a HIS tag. Purification was achieved by standard clarification of lysis, followed by two column chromatography steps demonstrating approximately 90-95% purity as measured by reverse phase liquid chromatography mass spectrometry. Endotoxin was determined to be ≤ 10 EU/mg.

## Treatment Groups and Regimen

### 3-Day Ischemic Cortical Stroke Acute Neuroprotection Screening Study

At the time of reperfusion following 60-min d-MCAO, mice were administered single doses of rhPDV constructs or equivalent volume of PBS vehicle control. Our rationale for the varying doses was based on previous preliminary in vitro data suggesting rhPDV_LG3_ might require 3 × the concentration of rhPDV_HEK293_ to achieve equivalent bioactivity [18]. Due to the conserved severity of this stroke (cortical-only), no special care beyond normal post-surgical monitoring is required. No functional outcome measures were assessed as the focus of the study was to evaluate only infarct volume. Subjects were monitored twice daily for health wellbeing and sacrificed 72 h post-reperfusion and brains assessed for infarct volume via TTC staining (see “Euthanasia, Brain Harvest, Tissue Sections, and TTC Staining”).

### Qualitative Assay of Blood–Brain Barrier Permeability

At the time of reperfusion following d-MCAO, 18 mice were randomly assigned to receive either rhPDV_LG3_ (6 mg/kg, IP) or PBS vehicle of equivalent volume (IP). Three subjects from each group were injected intraperitoneally with 4 mL/kg of 2% Evans blue in saline at 1 h, 20 h, and 44 h post-reperfusion. Four hours following administration of Evans blue at 5 h, 24 h, and 48 h post-reperfusion, dye circulation was confirmed visually (bluing of skin), and subjects were humanely euthanized via decapitation following the absence of pinch reflex under isoflurane anesthesia, as previously reported [17]. Whole brains were taken and briefly washed in PBS before being imaged. Brains were then placed in a slice matrix, and 1 mm coronal sections were taken and imaged. Visual inspection for extravasated dye (blue tissue) was performed to investigate potential gross alterations in BBB permeability following d-MCAO with or without rhPDV_LG3_ treatment. Although unforeseen logistical issues prevented transcardial perfusion of subjects and quantitative analysis of dye permeation, the qualitative results provide significant insight into the mechanisms underlying rhPDV_LG3_’s observed neuroprotection.

### Seven-Day Severe Ischemic Stroke Recovery Study

**Fig. 1:**
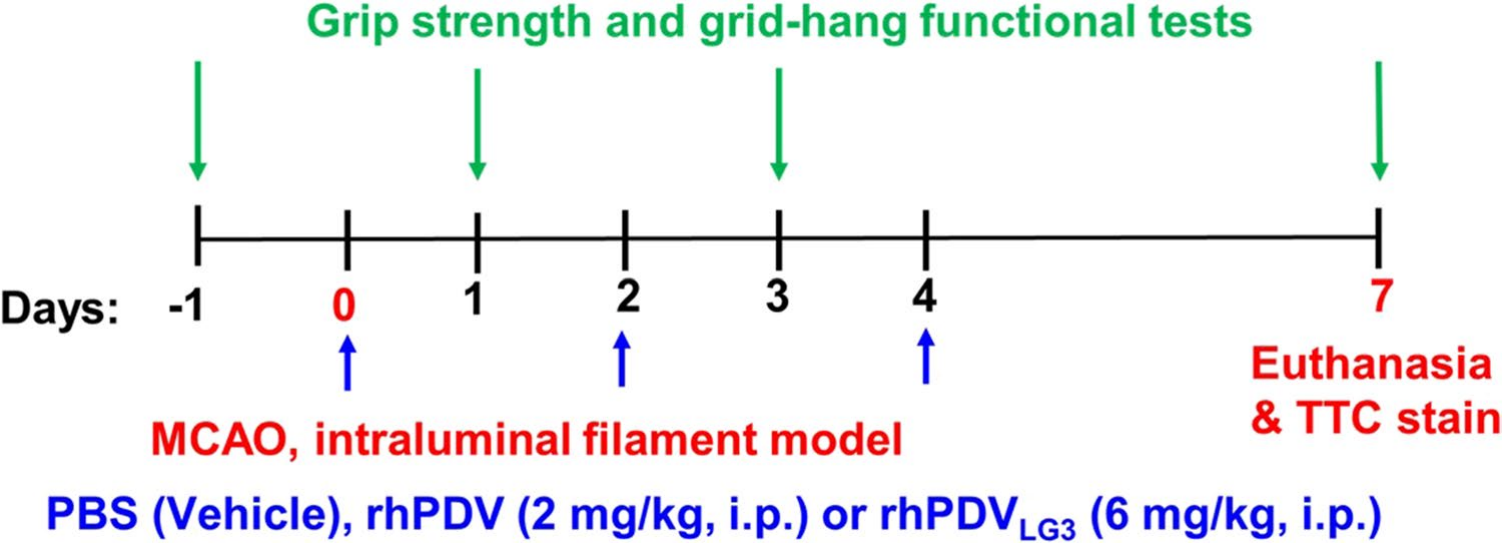
Protocol timeline to determine the neuroprotective effect of rhPDV (2 mg/kg) and rhPDV_LG3_ (6 mg/kg) following severe ischemic stroke

At the time of reperfusion following 60-min intraluminal t-MCAO, mice were administered doses of rhPDV_HEK293_ (2 mg/kg, positive control), rhPDV_LG3_ (6 mg/kg), or equivalent volume of PBS vehicle control, as well as on post-stroke days 2 and 4 (Fig. 1).

### Functional Outcome Assessments

All surviving mice were subjected to functional tests on days − 1 (baseline), 1, 3, and 7 post-surgery to evaluate changes in motor coordination and muscle strength by use of the grid-hang and grip strength tests. Prior to the commencement of each functional test, mice were allowed to acclimate to the test room for 1 h on each testing day.

#### Grid-Hang Test

A grid-hang test is a four-limb inverted hang test used to evaluate changes in neuromuscular impairment and motor coordination following ischemic stroke in mice. On days 1, 3, and 7 post-t-MCAO, all mice were individually placed at the center of a metal grid (lid of the home cage) and allowed to walk for 60 s, while the grid was inverted, approximately 25 cm from the table surface. The grid was cleaned with 70% ethanol prior to testing each mouse. The performance of mice was quantified and scored according to Table 1.

**Table 1:** Grid-hang test scoring criteria. All animals, as indicated, were assigned a range of scores from 0 to 2 based on the severity of deficit

Scoring criteria	Deficit score
*Normal*: mouse moved on the grid while holding onto metal dowels for 60 s with no paw slip and loss of grip	0
*Slight deficit*: mouse moved on the grid with paw slips while holding onto metal dowels for 60 s	0.5
*Partial deficit*: mouse moved on the grid with paw slips while holding onto metal dowels for 45 < 60 s before losing grip and falling off the grid	1.0
*Significant deficit*: mouse moved on the grid with paw slips while holding onto metal dowels for 30 ≤ 45 s before losing grip and falling off the grid	1.5
*Severe deficit*: mouse moved on the grid while holding onto metal dowels for 15 ≤ 30 s before losing grip and falling off the grid	1.75
*Complete deficit*: mouse moved on the grid while holding onto metal dowels for < 15 s before losing grip and falling off the grid	2.0

#### Grip Strength Test

A grip strength test is used to evaluate forelimb dysfunction and muscle strength after neurological impairment in rodents. The forepaw of each mouse was placed on the pull bar of the grip strength meter (Columbus Instruments, USA) by gently lifting each mouse by the tail. With relative consistency and uniform force across all trials, by the same experimenter, the base of the tail of each mouse was pulled towards the experimenter as soon as the torso of the mouse aligned horizontally to the pull bar and the measured value displayed on the monitor was recorded. An average of the grip strength values from three trials was taken per mouse on each testing day. Mice which failed to grip the pull bar with both paws were retested for that trial run. A zero score was assigned to any mouse which failed to grab onto the pull bar with both forepaws. Five minutes of rest was allowed between each trial, per mouse.

### Euthanasia, Brain Harvest, Tissue Sections, and TTC Staining

At day 3 (construct screening) or day 7 (stroke recovery study) post-stroke, all mice were humanely sacrificed via decapitation following the absence of pinch hindlimb reflex under isoflurane anesthesia. The whole brain was harvested and rinsed in cold PBS and placed in a 1-mm sectioning matrix (Kent Scientific Corporation, USA). Coronal slices of brain samples were incubated in pre-warmed 1% 2,3,5-triphenyltetrazolium chloride (TTC, Sigma, USA) for a total of 30 min (15 min per side). Brain slices were then sorted in a rostro-caudal manner for image acquisition using a 600-dpi resolution (HP scanner, G4050, USA). ImageJ 1.80 (NIH, USA) was used to process individual brain slices for the infarct area. Infarct volume is calculated by multiplying the summed slice area by the slice thickness. Ipsilateral and contralateral hemisphere areas were measured, and Swanson’s edema correction formulae were applied to adjust infarct volume of each slice. These volumes were summed for corrected infarct volume. The infarct volume is expressed as a percentage of the total brain volume (i.e., sum of ipsilateral and contralateral hemispheric volumes).

### Blinding Consideration and Statistical Analysis

All experimenters (involved in stroke induction, functional test, brain infarct volume analysis, and data analysis) were blind to treatment identity. All treatment intervention vials were labeled as a singular alphabetic letter per treatment group which were only revealed after completion of data analysis. Prior to treatment administration, two experimenters were present during treatment preparation for verification of blinded treatment assignments.

Data were analyzed, by an independent statistician, using GraphPad Prism 9.3 (GraphPad Software, USA) and are presented as mean ± SEM, where indicated in the figures. One-way ANOVA along with Tukey’s multiple comparisons test was used to analyze brain infarct volume. Repeated measures two-way ANOVA followed by Tukey’s multiple comparisons test was used to analyze body weight, grid-hang, and grip strength tests. The Mantel–Cox test was used to analyze differences in survival distribution among groups. Statistical significance of differences between groups was set at *p* < 0.05.

## Results

### Perlecan Domain V Constructs Provide Neuroprotection Following d-MCAO Stroke in Mice

Treatment effect on brain infarct volume was assessed in all treatment groups. Infarct volume as a percentage of total brain volume is presented in the left-center column (Table 2). Neuroprotection as percent reduction in infarct volume compared to PBS vehicle control is shown in the right-center column. Statistical analysis of significance between treatment groups’ results vs PBS vehicle control as shown by one-way ANOVA is displayed in the right column. Reference standard (rhPDV_HEK293_) significantly reduced infarct volume at 1 mg/kg (*p* = 0.0257) and 2 mg/kg (*p* < 0.0001) doses. rhPDV_Gly_ and rhPDV_NQ_ both significantly reduced infarct volume at 2 mg/kg (*p* = 0.0002, *p* = 0.005, respectively). rhPDV_LG3_ did not significantly reduce infarct volume at 2 mg/kg (*p* = 0.0872) but displayed a profound dose–response (see the inset figure) and conveyed significant protection at 4 mg/kg (*p* = 0.001) and 6 mg/kg (*p* < 0.0001) doses. These findings demonstrate that rhPDV_LG3_ is capable of matching or exceeding rhPDV’s level of neuroprotection and provides the first evidence of structure–activity relationship in perlecan domain V constructs’ neuroprotective efficacy following transient cortical stroke in mice.

**Table 2:**
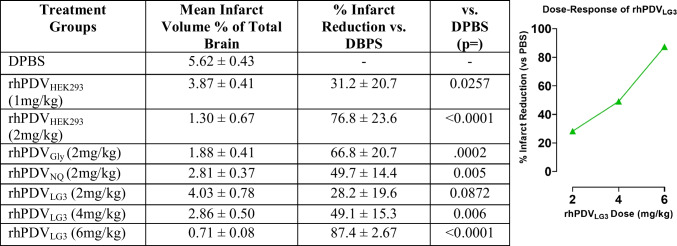
Variation in perlecan DV constructs conveys differential neuroprotection in d-MCAO stroke in mice. rhPDV-based constructs were screened for neuroprotective efficacy as measured by TTC-stained infarct volume 72 h post-reperfusion. Data presented is infarct volume as a % of total brain volume and neuroprotective efficacy % of control-PBS infarct volume reduction; *n* = 5–7 per group. The inset graph represents the dose–response curve expressed as % infarct reduction observed for rhPDV_LG3_

### rhPDVLG3 Appears to Modulate BBB Permeability Following Transient d-MCAO Stroke in Mice

**Fig. 2:**
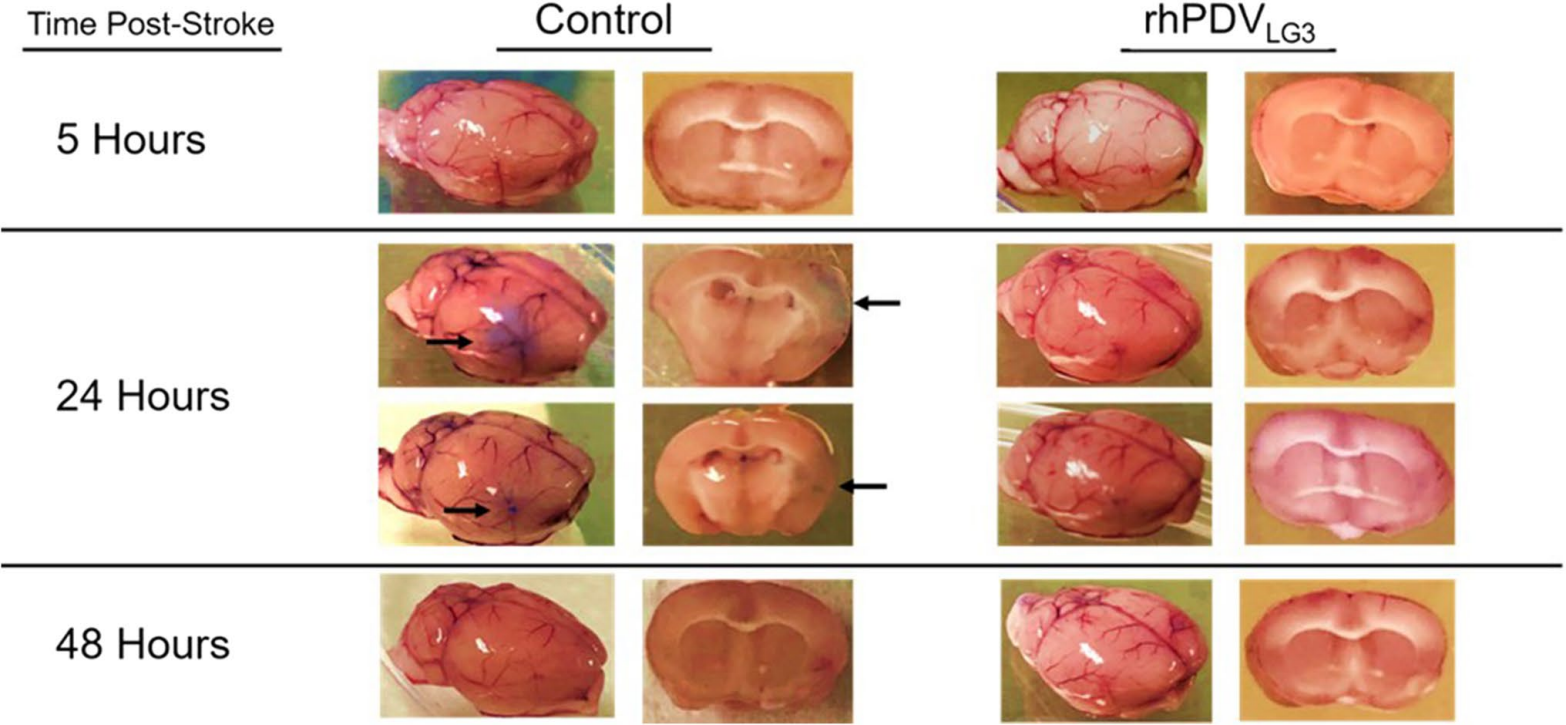
rhPDV_LG3_ inhibits dye permeation across the blood–brain barrier following d-MCAO stroke in mice. Representative color-saturated images of BBB permeation following stroke. Following d-MCAO stroke and treatment with either PBS or rhPDV_LG3_ (6 mg/kg, IP) at the time of reperfusion, changes to BBB integrity were investigated via Evan’s blue permeation at hours 5, 24, and 48 post-stroke, preventing dye permeation at all time points examined. Administration of rhPDV_LG3_ was found to inhibit the increased permeability observed in PBS-treated subjects 24 h post-stroke. No difference was visually observed between groups at 5- or 48-h time points; *n* = 4/group

Visualization of Evan’s blue dye permeation within brain tissue at hours 5, 24, and 48 post-stroke revealed significant alterations in PBS vehicle-treated control animals at 24 h post-stroke (Fig. 2 and Table 2). Varying levels of dye were observed in the significantly blued tissue of the distal MCA vascular territory (where d-MCAO stroke infarct occurs) of control subjects at 24 h, whereas little to no dye was observed in the brains of rhPDV_LG3_-treated subjects at that time point. Furthermore, increased BBB permeability as visualized by dye permeation in brain tissue was not observed in any subjects at 5- or 48-h time points. This suggests that rhPDV_LG3_ treatment at reperfusion leads to enhanced BBB integrity and that the first 24 h following d-MCAO may contain a critical therapeutic window for interventions aimed at neuroprotection via modulation/support of the BBB. Although quantification of dye permeation was not possible in this study, post-stroke BBB dynamics and potential interaction of rhPDV_LG3_ in BBB maintenance and recovery are key subjects of ongoing research.

### rhPDV_LG3_ Affords Superior Infarct Volume Reduction Than rhPDV Treatment Following Severe MCAO

**Fig. 3:**
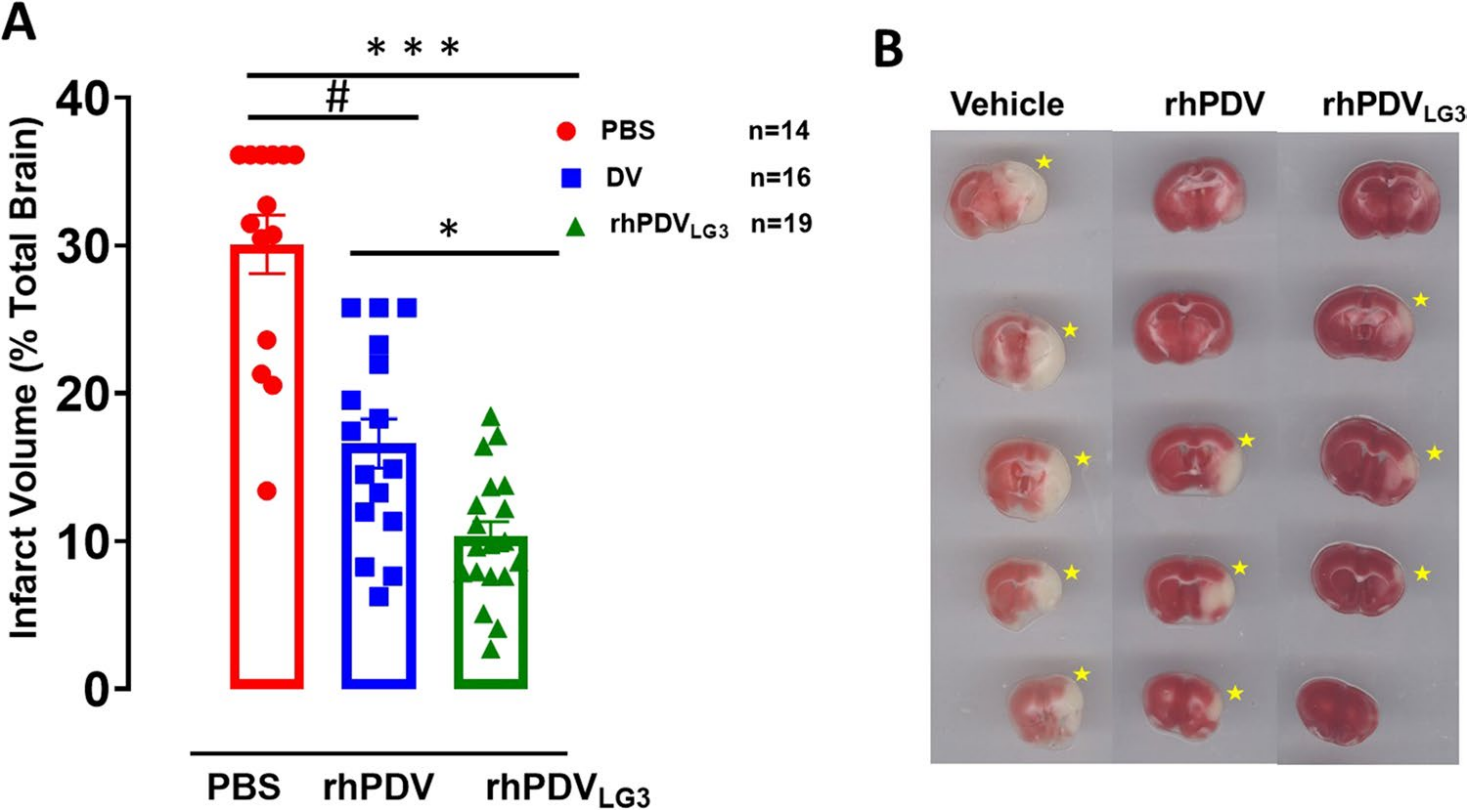
rhPDV_LG3_ reduces infarct volume following severe MCAO. **A** Infarct volume, 7 days following 60-min transient proximal MCAO (***p = 0.0001, ^#^*p* = 0.0001, *p = 0.0108, one-way ANOVA with multiple comparisons). **B** Representative brain slices, per treatment group, following 1% TTC staining on post-stroke day 7. Yellow asterisks indicate the infarcted hemisphere per slice. rhPDV and rhPDV_LG3_ were administered IP at 2 mg/kg and 6 mg/kg, respectively. Data presented as mean ± SEM

Treatment effect on brain infarct volume was assessed in all three groups. There was a statistically significant difference between treatment groups as shown by one-way ANOVA (*F*(2,46) = 42.70, *p* = 0.0001) (Fig. 3A). rhPDV_LG3_ significantly reduced infarct volume (65.6%) on post-t-MCAO day 7 when compared to rhPDV (44.7%)- and PBS-treated groups (Fig. 3B). Tukey’s multiple comparisons revealed that rhPDV_LG3_ significantly reduced infarct volume on post-t-MCAO day 7 when compared to rhPDV (*p* = 0.0108)- and PBS-treated group (*p* = 0.0001). Moreover, rhPDV significantly reduced brain infarct volume when compared to PBS-treated group (*p* = 0.0001). Thus, while rhPDV substantially reduced infarct volume when compared to PBS-treated control, rhPDV_LG3_ is superior to rhPDV in reducing infarct volume on post-stroke day 7 (Fig. 3A and 3B).

### rhPDV_LG3_ Improves Body Weight and Functional Outcomes and Prevents Mortality Following Severe MCAO

**Fig. 4:**
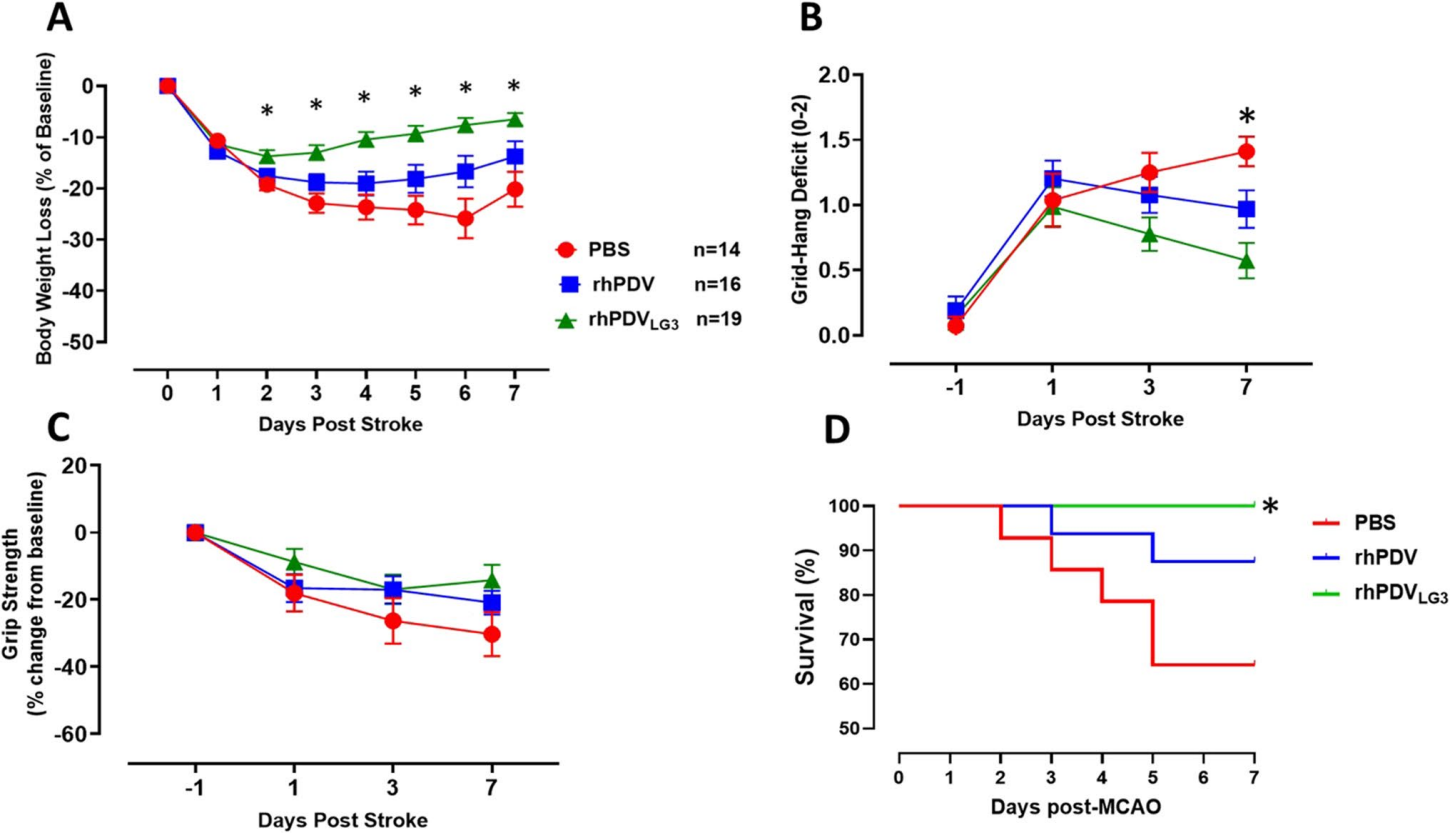
rhPDV_LG3_ improves body weight recovery and functional outcomes and reduces post-stroke mortality. **A** rhPDV_LG3_ (6 mg/kg) ameliorated body weight loss and enhanced body weight recovery following severe MCAO when compared to rhPDV (2 mg/kg) and PBS groups (*p < 0.05, two-way ANOVA with multiple comparisons). Body weight was measured daily and expressed as a percentage change from baseline (pre-MCAO) body weight. **B** Mice treated with rhPDV_LG3_ showed reduced grid-hang deficit on all measurement time points when compared to DV and vehicle groups (*p < 0.05, repeated measures two-way ANOVA with multiple comparisons). **C** rhPDV and rhPDV_LG3_ showed a trend for improved grip strength following severe MCAO when compared to the vehicle-treated group. **D** rhPDV_LG3_ prevented post-stroke mortality when compared to DV- and PBS-treated groups (*p* < 0.040, logrank test for trend). Data presented as mean ± SEM

Body weight was measured daily for all mice starting prior to t-MCAO surgery (at baseline) until euthanasia for evaluation of welfare. One day after t-MCAO, all mice regardless of treatment intervention presented with > 10% body weight reduction (Fig. 4A). However, there was a statistically significant interaction between treatment groups and post-stroke days as demonstrated by two-way ANOVA (*F*(14, 314) = 7.852, *p* = 0.0001). From post-stroke day 2 until euthanasia on post-stroke day 7, the rhPDV_LG3_-treated group steadily improved body weight measures with statistically significant differences on post-stroke day 2 (*p* = 0.0057), day 3 (*p* = 0.0009), day 4 (*p* = 0.0003), day 5 (*p* = 0.0004), day 6 (*p* = 0.001), and day 7 (0.0044) when compared to PBS-treated mice. There were statistically significant differences in the body weight of rhPDV_LG3_-treated mice on post-stroke day 3 (*p* = 0.03), day 4 (*p* = 0.0113), day 5 (*p* = 0.025), and day 6 (*p* = 0.0348) when compared to rhPDV-treated mice. There was no statistically significant difference (*p* > 0.05) between rhPDV- and PBS-treated groups at all time points post-stroke.

We evaluated grid-hang deficit (muscle coordination) and grip strength (forelimb muscle strength) at baseline and post-t-MCAO days 1, 3, and 7. There was a statistically significant interaction between treatment groups and post-stroke days for grid-hang deficit as revealed by two-way ANOVA (*F*(6, 136) = 3.109, *p* = 0.0069) (Fig. 4B). Mice treated with rhPDV_LG3_ had a statistically significant improvement in grid-hang deficit on post-stroke day 7 (*p* = 0.0001) when compared to PBS-treated mice. Also, Tukey’s multiple comparisons test reveals that rhPDV_LG3_ significantly improves grid-hang deficit on post-stroke day 7 when compared to PBS (*p* = 0.0001). However, there was no statistical difference between rhPDV_LG3_ and rhPDV at all measured time points (*p* > 0.05). Similarly, there was no statistical difference between rhPDV- and PBS-treated groups at all time points (*p* > 0.05).

Although mean grip strength values for rhPDV_LG3_ trended towards improvement when compared to the two other treatment groups, there was no statistically significant interaction between groups and post-stroke days as demonstrated by two-way ANOVA (*F*(6, 138) = 1.545, *p* = 0.1680). Mice treated with rhPDV_LG3_ did not significantly improve grip strength measures when compared to rhPDV- and PBS-treated groups at all measured time points (*p* > 0.05) (Fig. 4C). This is consistent with other studies that have shown statistically significant increases in grip strength not manifesting until after PSD7.

Across all treatment groups, 14% of animals died before post-MCAO day 7. At all experimental time points following transient MCAO induction, the rhPDV_LG3_-treated group showed 100% survival (Fig. 4D). However, mortality in the rhPDV-treated group was 12.5% after MCAO induction. Specifically, 6.25% mortality occurred on day 3 and day 5 post-t-MCAO. That is, one mouse was found dead in the cage on each of these 2 days, among the rhPDV treatment group. There was a total of 35.7% mortality among the PBS (vehicle)-treated mice following t-MCAO. Approximately 7.1% mortality occurred among the PBS-treated group on post-MCAO days 2, 3, and 4, while 14.2% mortality was recorded on post-t-MCAO day 5 alone. The difference in mortality curves between groups was statistically significant (*p* = 0.0040). This indicates that rhPDV_LG3_ prevented post-stroke mortality when compared to moderate mortality in the rhPDV treatment group, with the highest mortality occurring in the vehicle-treated group (Fig. 4D).

Only one mouse from the PBS-treated group was euthanized on post-stroke day 1 for welfare concerns. Hence, for statistical analyses of mortality and survival rate, a total of 14 mice were assumed for the PBS treatment group after the euthanized mouse was excluded.

## Discussion

These studies provide the first empirical evidence to suggest that rhPDV_LG3_ (6 mg/kg, IP), a subdomain of perlecan DV, provides enhanced life-saving and neuroprotective benefits over rhPDV (2 mg/kg, IP) resulting in reduced mortality, improved body mass retention and recovery, and improved acute functional recovery following severe experimental ischemic stroke in male mice survived for 7 days. Although the present findings show that rhPDV remains active in reducing infarct volume when compared to vehicle-treated controls, as previously demonstrated in less severe experimental stroke models [7, 8, 13], these data further corroborate the hypothesis that (6 mg/kg dosage of) rhPDV_LG3_, a 21-kD peptide occupying the c-terminus of rhPDV which binds more avidly to the α2β1 integrin receptor, may ultimately be superior to rhPDV (treatment at 2 mg/kg) as a therapeutic agent for acute ischemic stroke.

As a major component of the cerebral microvasculature ECM contributing to BBB integrity, perlecan DV is rapidly degraded within 2 h of stroke injury [11]. Similar to perlecan DV, the c-terminal laminin-like domain, LG3, is proteolytically cleaved by cysteine proteases [19, 20]. Importantly, soluble LG3 has been identified in the blood and urine where elevated levels are reported to correlate with increased physical (aerobic) activity [21, 22], suggesting this molecule may serve an integral role supporting homeostatic function of the CNS.

In vitro reports on the mechanistic insight of perlecan DV’s neuroprotective and neuro-angiogenic roles in hypoxic conditions hold that perlecan DV increases VEGF secretion by communicating with α5β1 integrin through the diversity-generating retroelement (DGR) amino acid sequence biding motif [13, 23]. One of the reports found that LG3 also binds to α5β1 integrin, nevertheless with lower affinity than parent perlecan DV [23]. The rationale to compare the biologic action of these molecules in the present study at different doses relates to differences reported for receptor saturation kinetics and possible differences in preferred ligands, alluded to the above. To this end, our preliminary screening studies surprisingly demonstrated equivocal results with respect to inhibition of infarct volume for rhPDV_LG3_ and rhPDV_HEK293_ administered at 6 mg/kg and 2 mg/kg, respectively. Furthermore, rhPDV_LG3_ exhibited a tight dose–response correlation with respect to neuroprotective activity in the d-MCAO stroke model in mice. Based on the results of our construct screening work, rhPDV_LG3_ was selected as the lead construct for thorough investigation of neuroprotection and acute functional recovery in the more severe intraluminal filament t-MCAO stroke model. Surprisingly, based upon the present findings, rhPDV_LG3_ has the potential to reduce brain infarct volume and reduce mortality to a greater extent post-stroke than its parent molecule.

Neurons consume approximately 75–80% of the total energy produced within the brain [24, 25]. Consequently, during catastrophic failure of oxygen supply, such as in the case of LVO stroke, the body is unable to compensate once endothelial dysfunction has progressed to a stage at which BBB disruption has been initiated. In this context, administration of supraphysiologic levels of rhPDV has been found to reduce BBB dysfunction while promoting BBB repair by inducing the migration and proliferation of platelet-derived growth factor receptor β (PDGFRβ) positive pericytes to the ischemic brain following experimental stroke; Nakamura et al. [26] aptly demonstrated that rhPDV maintains the BBB by its simultaneous interaction with α_5_β_1_ integrin and PDGFRβ to regulate focal adhesion and actin cytoskeleton in the ischemic tissue. After stroke, the increased expression of PDGFRβ is vital for the restoration of the microvasculature and BBB function, as well as for neuro-reparative cascades within the ischemic core region [9, 26–28]. Indeed, the role of pericyte recruitment in supporting capillary morphogenesis and stabilization of developing microvascular networks has been described as a critical step during embryonic development and in the restoration of capillary dysfunction in health and disease [29–32]. We hypothesize, therefore, that perlecan DV, and more directly LG3, functions at the molecular level to guide pericyte recruitment to areas of BBB disruption, minimizing neuronal death once oxygen delivery has been restored through capillary morphogenesis. Indeed, assays utilizing Evan’s blue injections to visualize BBB permeability following stroke demonstrated a robust restorative effect of rhPDV_LG3_ on BBB integrity at 24 h post-stroke compared PBS-treated controls (Fig. 1). Notably, Nakamura and colleagues [26] reported a moderate protective effect of rhPDV treatment with respect to infarct volume in WT control mice receiving rhPDV 24 h after intraluminal filament MCAO relative to rhPDV treatment of perlecan deficient mice. Our present findings differ from their report with respect to the magnitude of rhPDV’s neuroprotective action in WT mice, which may be directly related to the time of initiation of therapeutic treatment.

We show a statistically significant reduction (*p* = 0.0001) in infarct volume by rhPDV treatment as compared to vehicle control using a similar ischemic stroke model and route of administration (IP injection); importantly however, our therapeutic regimen was initiated immediately upon reperfusion. It is well accepted that significant disruption of the BBB occurs within 24 h following reperfusion in untreated mice subjected to proximal MCAO which is directly associated with increased infarct volume [33, 34]. This suggests that substantial reduction in brain infarct volume observed in the present study (65.6% and 44.7% for rhPDV_LG3_ and rhPDV, respectively) is mediated by the timely administration of drug at reperfusion and that a 24 h delay in treatment delivery in this model of severe ischemic stroke may not be as effective given that reperfusion evokes significant injury related to increased production of reactive oxygen species. Bix lab previously reported, albeit in a photothrombotic model of ischemic stroke, that early administration of rhPDV at 3 h following surgery significantly reduces infarct volume when compared to initial rhPDV treatments started at 6, 12, and 24 h after stroke induction [7]. The authors further showed that brain infarct volume is proportional to the time delay in rhPDV administration [7]. Hence, early administration of rhPDV_LG3_ (and rhPDV) in this model of severe ischemic stroke is crucial to reduce BBB disruption and brain infarct volume.

Several key pieces of evidence support a putative mechanism by which rhPDV_LG3_ exerts the observed neuroprotective actions following stroke. rhPDV_LG3_ initiates robust anti-apoptotic responses in both mesenchymal stem cells and cultured fibroblasts [35], in addition to promoting vascular smooth muscle cell migration [36] through direct interaction with α2β1 integrin. Anti-apoptotic action following LG3 administration is reported to occur downstream of ERK1/2. The potential for this pathway to be active within the functional NVU following acute cerebral ischemia, specifically contributing to the survival of one or more cell types (astrocytes, neurons, pericytes, or neuroprogenitor cells), has to date not been explored; however, indirect evidence suggests function blocking antibodies directed towards the β1 integrin subunit interrupt BBB restoration following intravascular administration immediately prior to stroke in mice [37]. Willis et al. [38] have successfully demonstrated two distinct biological functions for two distinct domains of perlecan DV (endorepellin) which can be functionally dissociated by treatment with a blocking monoclonal antibody targeting the I domain of the α2 integrin subunit, providing direct proof as to LG3’s preference for utilization of the α2β1 integrin signaling pathway. Future studies will seek to understand whether α2β1 integrin/SFK (Src and Fyn)/PI3K- or ERK1/2-dependent pathways can be demonstrated to mediate the anti-apoptotic activity of rhPDV_LG3_ which may in part contribute to the neuroprotective action of rhPDV_LG3_.

While the recent adoption of endovascular therapies has significantly reduced stroke burden associated with LVO stroke by > 40% [39], an opportunity exists to further reduce this statistic as well as persistent post-stroke morbidity through application of novel neuroprotective therapies that can be administered either prior to or immediately upon reperfusion [40]. Herein, we demonstrate for the first time that rhPDV_LG3_ may fulfill a great unmet stroke therapy need, an outcome to be investigated in future clinical trials. The finding of complete protection from mortality (in male mice) was an unexpected finding as the experimental intraluminal filament model of ischemic stroke often results in high (15–30%) mortality [41].

An important observation made during the in-life portion of the filament MCAO study which may be predictive of mortality risk and severity of post-stroke physical disability was the acute loss of body mass for animals of the control group that occurred within 24 h (− 11.39 ± 0.72%) post-stroke and which peaked from PSD3-PSD5 (− 22.0–22.5% ± 2.84). Our findings corroborate those reported by Springer et al. in which young male mice were similarly subjected to severe 60 min MCAO [42]. In that study, the authors reported that catabolic wasting of muscle and fat separately accounted for 12% and 27% of loss in total body mass, respectively, and that infarct volume correlated with severity of muscle wasting. Importantly, interventions addressing potential mechanisms regulating muscle wasting in that study, including high caloric intake, β-receptor blockade, and antibiotic treatment, failed to reverse acute loss in body mass resulting from severe stroke. Furthermore, the temporal delay in body weight recovery (approximately 3 days) for animals receiving 2 mg/kg of parent rhPDV in contrast to 6 mg/kg rhPDV_LG3_ may provide additional insight into possible mechanisms regulating muscle wasting following stroke injury. Assuming there is no difference in the rate at which these two molecules have been observed to home to the penumbra [13, 23] and recognizing that LG3 is released from parent perlecan DV through the action of focally expressed cathepsin activity [19, 20, 43], we speculate that the administration of 2 mg/kg rhPDV dose to stroke patients may produce an unnecessary delay in neuroprotective action.

Severe loss of body mass is a common consequence of stroke in adults and is predictive of decreased physical and functional outcome [44, 45]. Results from the present study demonstrate a significant improvement in body weight recovery and in grid-hang performance following administration of rhPDV_LG3_, suggesting a powerful effect on intracellular signaling associated with neuronal loss and post-stroke cachexia impacting physical activity. Critically, rhPDV_LG3_-treated subjects began improvements from PSD1 to PSD3, reversing the down-ward trend observed in rhPDV and vehicle controls. Grid-hang deficit as employed in the present study is a combined modification of the foot-fault and wire-hang test for the purpose of simultaneously evaluating motor coordination and grip strength in mice following stroke. The advantage of our modified grid-hang deficit test is the time measured ability of each mouse to coordinate movement using all four limbs and their ability to grasp onto the grid without falling off within 60 s. While our previous reports on rhPDV did not use this functional test per se, we had demonstrated that mice receiving rhPDV exhibited a significant improvement in the number of recorded foot-faults compared to control mice [7, 8]. rhPDV treatment trended towards an improvement, although not statistically significant, on PSD3 and PSD7 compared to the vehicle-treated group.

We did not observe any significant difference in grip strength measured between groups at any time point. However, we observed a moderate increase in grip strength on PSD3 and PSD7 in rhPDV_LG3_-treated mice compared to the vehicle-treated group rhPDV treatment resulted in a similar modest increase at PSD3, but mean performance trended down to PSD7-contrasting the reversed trajectory observed in rhPDV_LG3_-treated subjects from PSD3 to PSD7. This contrast reflects observations in body weight recovery and grid-hang performance, where rhPDV_LG3_-treated subject’s mean body weight recovery began 48hrs earlier than those treated with rhPDV. One explanation for this difference could be that rhPDV ineffectively affords acute neuroprotection (compared to rhPDV_LG3_), as explained earlier, and therefore, grip strength was measured too early following stroke to detect meaningful improvement. Other studies have indicated grip strength differences between treated and control animals well beyond, but not prior to, PSD7 in photothrombotic and distal MCAO models [8, 46, 47]. In fact, a recent study from the Bix Lab showed that rhPDV, when compared to vehicle, increased grip strength following distal MCAO after PSD8 [8]. Since the neurological severity of the stroke model used in the current study is greater than that in our previous study, we expected to observe statistically significant difference between groups. We assessed grip strength as a measure of stroke-induced changes in forelimb muscle strength which models an important clinical outcome measure of upper limb function in patients [48, 49]. Although it is not uncommon to assess limb strength individually [50, 51], we chose to measure muscle strength in both limbs at the same time rather than individually to forestall laterality bias in the values of each measure. Overall, rhPDV_LG3_ substantially improved motor function 7 days after severe ischemic stroke, while rhPDV trended, albeit statistically insignificantly, towards improved measures of functional outcome compared to the vehicle-treated group.

Ischemic stroke is a particularly challenging condition to model due to heterogeneity in location, severity, and patient population. Even within preclinical stroke models, intragroup variation persists and can often diminish the ability to detect differences between groups. Here, we modeled LVO (proximal and distal MCAO) and provide substantial evidence for therapeutic bioactivity of rhPDV-based therapies in the form of neuroprotection and, in the case of proximal MCAO, metabolic/physiologic homeostasis and acute functional recovery. In accordance with the ARRIVE and IMPROVE guidelines [14, 52], our study will benefit from the inclusion of considerations for sex, age, and comorbidities such as hypertension and diabetes mellitus as relevant translational factors present in clinical ischemic stroke populations. Additionally, the inclusion of histological assessment of brain morphology would have increased the scope and depth of our investigation of the mechanisms underlying rhPDV_LG3_ and rhPDV’s robust neuroprotective effect. However, addressing these limitations is the subject of ongoing investigations in our lab as the present study has provided answers to the novel questions which satisfy the premise of our hypothesis. Nevertheless, the strength of the present study is our strict criterion for stroke induction/subject inclusion, randomization of subjects to groups, and blinding to treatment groups at all stages until study completion. These criteria were ensured to forestall experimental bias during MCAO induction, group treatments, and data analysis.

In conclusion, we demonstrated that rhPDV_LG3_ is neuroprotective and acutely functionally restorative, enhances body weight retention and recovery, and reduces mortality in a severe experimental ischemic stroke model in male mice. Also, we show the first evidence which suggests that 6 mg/kg of rhPDV_LG3_ may be more effective than 2 mg/kg of rhPDV in the context of ischemic stroke with respect to temporal kinetics of neuroprotection and is deserving of further translational evaluation as a potential therapy supporting stroke recovery.

## Data Availability

The corresponding author will make supporting data available upon request.
